# Tumor-Specific Imaging with Angiostamp800 or Bevacizumab-IRDye 800CW Improves Fluorescence-Guided Surgery over Indocyanine Green in Peritoneal Carcinomatosis

**DOI:** 10.3390/biomedicines10051059

**Published:** 2022-05-03

**Authors:** Véronique Josserand, Claire Bernard, Thierry Michy, Mélanie Guidetti, Julien Vollaire, Jean-Luc Coll, Amandine Hurbin

**Affiliations:** 1 Institute for Advanced Biosciences, Institut National de la Santé et de la Recherche Médicale INSERM U1209, Centre National de la Recherche Scientifique CNRS UMR5309, Université Grenoble Alpes, F-38000 Grenoble, France; veronique.josserand@univ-grenoble-alpes.fr (V.J.); claire.bernard@chu-brest.fr (C.B.); tmichy@chu-grenoble.fr (T.M.); mguidetti@synapcell.fr (M.G.); julien.vollaire@univ-grenoble-alpes.fr (J.V.); jean-luc.coll@univ-grenoble-alpes.fr (J.-L.C.); 2 Centre Hospitalier Universitaire Grenoble Alpes, Université Grenoble Alpes, F-38000 Grenoble, France

**Keywords:** fluorescence-guided surgery, peritoneal carcinomatosis, indocyanine green, Bevacizumab-IRDye 800CW, Angiostamp800, bioluminescence imaging, neoadjuvant chemotherapy

## Abstract

Complete surgical removal of lesions improves survival of peritoneal carcinomatosis and can be enhanced by intraoperative near-infrared fluorescence imaging. Indocyanine green (ICG) is the only near-infrared fluorescent dye approved for clinical use, but it lacks specificity for tumor cells, highlighting the need for tumor-selective targeting agents. We compared the tumor-specific near-infrared fluorescent probes Bevacizumab-IRDye 800CW and Angiostamp800, which target tumor angiogenesis and cancer cells, to ICG for fluorescence-guided surgery in peritoneal carcinomatosis of ovarian origin. The probes were administered to mice with orthotopic peritoneal carcinomatosis prior to conventional and fluorescence-guided surgery. The influence of neoadjuvant chemotherapy was also assessed. Conventional surgery removed 88.0 ± 1.2% of the total tumor load in mice. Fluorescence-guided surgery allowed the resection of additional nodules, enhancing the total tumor burden resection by 9.8 ± 0.7%, 8.5 ± 0.8%, and 3.9 ± 1.2% with Angiostamp800, Bevacizumab-IRDye 800CW and ICG, respectively. Interestingly, among the resected nodules, 15% were false-positive with ICG, compared to only 1.4% with Angiostamp800 and 3.5% with Bevacizumab-IRDye 800CW. Furthermore, conventional surgery removed only 69.0 ± 3.9% of the total tumor burden after neoadjuvant chemotherapy. Fluorescence-guided surgery with Angiostamp800 and Bevacizumab-IRDye 800CW increased the total tumor burden resection to 88.7 ± 4.3%, whereas ICG did not improve surgery at all. Bevacizumab-IRDye 800CW and Angiostamp800 better detect ovarian tumors and metastases than the clinically used fluorescent tracer ICG, and can help surgeons completely remove tumors, especially after surgery neoadjuvant chemotherapy.

## 1. Introduction

Peritoneal carcinomatosis with widespread micrometastases frequently occurs in patients with advanced gynecological cancers and has a very poor prognosis [1]. Current treatment is based on cytoreductive surgery complemented by adjuvant platinum-based chemotherapy. Neoadjuvant chemotherapy can be performed in some cases to decrease tumor burden and improve the surgery. It is clearly demonstrated that complete surgical removal of macroscopic lesions improves overall and progression-free survival [1,2]. However, precise tumor localization is mainly intraoperative and relies on surgeon visual inspection and palpation to differentiate between benign and tumor lesions. Intraoperative near-infrared (NIR) fluorescence imaging allows for precise detection of tumors and excision margins thanks to limited tissue autofluorescence and absorption phenomena and may improve resection rate and thus positively influences prognosis [2,3,4,5].

Non-targeted contrast agents that passively accumulate in tumors and fluorescent probes targeting a receptor overexpressed in tumor cells or a tumor-related molecular event have been developed for intraoperative fluorescence-guided tumors surgery [3,5]. Indocyanine green (ICG) is the only NIR fluorescent probe approved for clinical use by the Food and Drug Administration (FDA) and the European Medicines Agency (EMA). After systemic injection, ICG circulates in the bloodstream and accumulates into tumor tissue due to the enhanced permeability and retention effect [6]. ICG has low toxicity and is mainly used for intraoperative angiography, detection of sentinel lymph nodes, and fluorescence-guided surgery of various cancers [6,7,8]. Studies using ICG for intraoperative imaging showed good visibility of peritoneal metastases [8,9,10]. However, a high false-positive rate was shown [9], which can be disabling. In addition, distinguishing between benign and malignant lesions became an issue when patients with ovarian cancer were treated with hyperthermic intraperitoneal chemotherapy [10]. The lack of specificity of ICG for tumor cells highlights the need for tumor-selective targeting agents.

Several tumor-specific ligands recognizing cancer-related molecular biomarkers have been evaluated for fluorescence imaging applications [3,5]. Bevacizumab is an antibody targeting tumors that overexpress vascular endothelial growth factor (VEGF), which is an important regulator of angiogenesis and vascular permeability [3]. Besides ICG, IRDye 800CW is the most commonly used NIR fluorescent dye in clinical settings [3,5]. The safety and feasibility of using Bevacizumab labeled with IRDye 800CW for tumor-specific optical imaging have already been shown in patients with primary breast cancer [11,12], esophageal cancer [13], colorectal adenoma [14], and peritoneal carcinomatosis of colorectal origin [15]. Fluorescence imaging with Bevacizumab-IRDye 800CW enabled in situ detection of additional peritoneal malignant lesions, while fluorescence uptake was absent in clinically suspicious but benign lesions [15]. Angiostamp800 specifically targets α_v_β_3_ integrin, which is overexpressed in tumor neoangiogenesis and aggressive migrating cancer cells [16]. NIR optical imaging using Angiostamp800 has been shown to provide a strong fluorescence signal in tumor tissue with low non-specific background, generating good tumor contrast in various animal cancer models, including fibrosarcoma [17], osteosarcoma [18], head and neck squamous cell carcinoma [19], bone metastasis [20], liver metastasis [21], and peritoneal carcinomatosis [22,23,24]. Despite promising results showing a more exhaustive surgery, with a decrease in resection margins and an increase in survival rate [17,18,19,22,23,24], this optical probe has not yet entered the clinical stage.

ICG, Bevacizumab-IRDye 800CW, and Angiostamp800 have thus shown potential for fluorescence-guided cancer surgery but have different levels of maturity in the clinic. In this study, we evaluated the potential of the anti-angiogenic targeted probes Bevacizumab-IRDye 800CW and Angiostamp800, compared with ICG, to detect ovarian cancer and multiple disseminated tumor nodules in a mouse model of peritoneal carcinomatosis of ovarian origin. This is the first time these three optical probes have been used in parallel under the same experimental conditions. This allowed us to clearly compare side-by-side their in vivo bio-distribution, pharmacokinetics, tumor specificity, and efficacy in improving tumor resection, including after neoadjuvant chemotherapy.

## 2. Materials and Methods

### 2.1. Fluorescent Probes and Drugs

Paclitaxel and carboplatin were obtained from Sigma-Aldrich (St Quentin-Fallavier, France). Bevacizumab-IRDye 800CW was provided by SurgVision (Groningen, The Netherlands) and formulated at 6.5 µmol/L (1 mg/mL) in an isotonic buffer at pH 7.0. Angiostamp^TM^800 (Fluoptics, Grenoble, France) was provided at 50 µmol/L in 1× PBS, and the clinical formulation of ICG (SERB, Paris, France) was solubilized at 14 mmol/L in DMSO and then diluted to 500 µmol/L in 1× PBS. The fluorescence properties of each probe are recapitulated in Supplementary Table S1.

Serial dilutions were prepared in murine plasma, and fluorescence signals of 10 μL samples were measured using the Fluobeam^®^800 (Fluoptics, Grenoble, France) and compared to the background signal of plasma. The detection limit was reached when the signal-to-background ratio reached 2.0.

### 2.2. Cell Lines

The human ovarian adenocarcinoma SKOV3 cell line was obtained from the LGC Standard (Molsheim, France) and maintained in culture at 37 °C in RPMI-1640 medium with 10% FBS in a 5% CO2 humidified atmosphere. SKOV3 cell line was authenticated using Short Tandem Repeat (STR) analysis (ATCC, Manassas, VA, USA). SKOV3-Luc cells stably expressing firefly luciferase were derived from SKOV3 cells [25] as described in Supplementary Methods [25,26,27,28,29,30].

### 2.3. In Vivo Tumor Models

All applicable institutional and/or national guidelines for the care and use of animals were followed. All animal experiments were performed in accordance with the institutional guidelines of the European Community (EU Directive 2010/63/EU) for the use of experimental animals and were approved by an ethic committee (Cometh38 Grenoble, France) and the French Ministry of Higher Education and Research under the reference: apafis#15176-201808281433752v1.

All procedures were performed under anesthesia (4% isoflurane/air for anesthesia induction and 1.5% after that) with six-week-old female NMRI nude mice (Janvier Labs, Le Genest-Saint Isle, France). Subcutaneous ovarian tumor model, pharmacokinetics studies on blood plasma samples, and in vivo biodistribution of fluorescent probes are described in Supplementary Methods.

Orthotopic peritoneal carcinomatosis of ovarian origin: Mice were injected intraperitoneally with 107 SKOV3-Luc cells in 400 µL 1× PBS. Tumor growth and peritoneal invasion were monitored weekly by in vivo bioluminescence imaging (IVIS Kinetic, Perkin Elmer, Waltham, MA, USA) 5 min after the intraperitoneal injection of 150 mg.kg^−1^ of D-luciferin (Promega, Charbonnière, France).

### 2.4. Fluorescence-Guided Surgery in Mice with Orthotopic Peritoneal Carcinomatosis

Mice with orthotopic peritoneal carcinomatosis (usually within 25–30 days after tumor cells implantation) were distributed into three comparable groups of mice based on noninvasive in vivo bioluminescence imaging evaluating preoperative tumor burden. Mice were anesthetized, and 1.6 mg/kg Angiostamp800 (10 nmol/mouse; *n* = 13 mice), or 3.5 mg/kg Bevacizumab-IRDye 800CW (0.65 nmol/mouse; *n* = 9 mice), or 4 mg/kg ICG (113 nmol/mouse; *n* = 11 mice) were administered intravenously via the tail vein by an investigator, who was the only person aware of the treatment group allocation. Sixteen hours after administration of Angiostamp800 or ICG and 96 h after administration of Bevacizumab-IRDye 800CW, mice received an intraperitoneal injection of 150 mg/kg of D-luciferin. Tumor resection was then performed in 2 steps by the surgeon, who was not informed of which probe was injected. First, a conventional laparotomy procedure was performed under normal light, including hysterectomy, bilateral adenectomy, and resection of all tumor nodules detectable by the surgeon’s naked eye. Then, cytoreduction was further completed under fluorescence guidance using the Fluobeam^®^800 (blinded fluorescence-guided surgery study). Finally, the mouse body was imaged by bioluminescence to assess the final tumor residues in mice after the surgery.

The same surgical procedure was applied three days after neoadjuvant chemothera-py in mice with established peritoneal carcinomatosis (*n* = 3 mice per group). Neoadjuvant chemotherapy consisted in two cures four days apart of paclitaxel (10 mg/kg administered intravenously) and carboplatin (40 mg/kg administered intraperitoneally).

### 2.5. Ex Vivo Surgical Specimen Examination by Sequential Bioluminescence and Fluorescence Imaging

All surgical specimens were placed on dedicated grids and sequentially submitted to ex vivo bioluminescence (IVIS Kinetic, Perkin Elmer, Waltham, MA, USA) and fluorescence (Fluobeam^®^800, Fluoptics, Grenoble, France) imaging. All specimens with negative or low bioluminescence levels (<5.10^5^ RLU/100 ms) were further analyzed for luciferase protein expression using the luciferase assay system (Promega, Charbonnière, France) following the manufacturer’s instructions. Samples with negative luciferase expression and positive fluorescence signal (higher than 2 times the background noise) were considered false positives.

The total tumor burden was calculated as the sum of bioluminescence signals from conventionally resected specimens, fluorescence-guided surgical specimens, and unresected tumor residues left in the mouse body after surgery.

### 2.6. Statistical Analyses

All analyses were performed using GraphPad Prism 8.4.3 software (GraphPad Software Inc., San Diego, CA, USA). Statistical comparisons between groups were conducted with the Kruskal–Wallis test with Dunn’s multiple comparisons post-hoc test. Statistical comparisons between mice groups over time were determined by two-way ANOVA with Tukey’s or Sidak’s post-hoc test. Correlations between two continuous variables were analyzed using Spearman’s rank correlation. Statistical significance was defined for *p* values ≤ 0.05. *, *p* < 0.05; **, *p* < 0.01; ***, *p* < 0.001.

## 3. Results

### 3.1. Bevacizumab-IRDye 800CW Circulated Longer in the Bloodstream than Angiostamp800 and ICG

Fluorescence signals of the probes were detected using the Fluobeam^®^800 device (Supplementary Figure S1). Detection sensitivity was related to the fluorescence molecular properties of the probes (Supplementary Table S1), resulting in different fluorescence signals.

We chose the doses used in vivo in mice for the three fluorescent probes based on the literature and our expertise: 3.5 mg/kg (113 nmoles/mouse) ICG [31], 4 mg/kg (0.65 nmoles/mouse) Bevacizumab-IRDye800CW [32,33], and 1.6 mg/kg (10 nmoles/mouse) Angiostamp800 [22,24]. After intravenous administration, both Angiostamp800 and ICG were rapidly cleared from the bloodstream (elimination half-lives: 30 min and 17 min, respectively) (Supplementary Figure S2). Conversely, Bevacizumab-IRDye 800CW was circulated for several days (elimination half-life: 34 h), as expected with an antibody, preventing intraoperative imaging in the early post-injection period. The best time to perform fluorescence imaging after probe injection was determined based on these data: 16 h for ICG and Angiostamp800, and 96 h for Bevacizumab-IRDye 800CW. This is the best compromise between fluorescence signal in the tumor and tumor specificity.

### 3.2. Angiostamp800, Bevacizumab-IRDye 800CW, and ICG Accumulated in Primary Ovarian Tumors

For the three fluorescent probes, in vivo and ex vivo fluorescence imaging demonstrated tumor uptake after intravenous injection in mice bearing subcutaneous SKOV3 ovarian tumors. Indeed, both ICG and Angiostamp800 showed an immediate fluorescence signal in the tumor followed by a gradual decrease up to 24 h (Supplementary Figure S3). In the meantime, ICG and Angiostamp800 were gradually eliminated by the liver and kidneys, respectively. In contrast, Bevacizumab-IRDye 800CW showed progressive tumor accumulation up to 24 h, followed by a slight decrease up to 96 h according to its pharmacokinetics (Supplementary Figure S3). Bevacizumab-IRDye 800CW also immediately accumulated in the liver, and the hepatic signal, although gradually decreasing, remained until 96 h. These data were confirmed by ex vivo fluorescence imaging on collected organs at 24 h post-injection for ICG and Angiostamp800 and 96 h post-injection for Bevacizumab-IRDye 800CW (Supplementary Figure S4a,b), showing a high tumor signal with good tumor specificity as illustrated by high tumor-to-muscle ratios (Supplementary Figure S4c). High signals were also observed in the eliminating organs, i.e., kidneys for Angiostamp800, liver for Bevacizumab-IRDye 800CW, and both kidneys and liver for ICG. Interestingly, only the kidneys showed a higher signal than the tumor with Angiostamp800, whereas residual fluorescence was observed to a slight extent in most organs with Bevacizumab-IRDye 800CW and ICG.

### 3.3. Angiostamp800 and Bevacizumab-IRDye 800CW Improved Fluorescence-Guided Surgery of Peritoneal Carcinomatosis More than ICG

**Figure 1:**
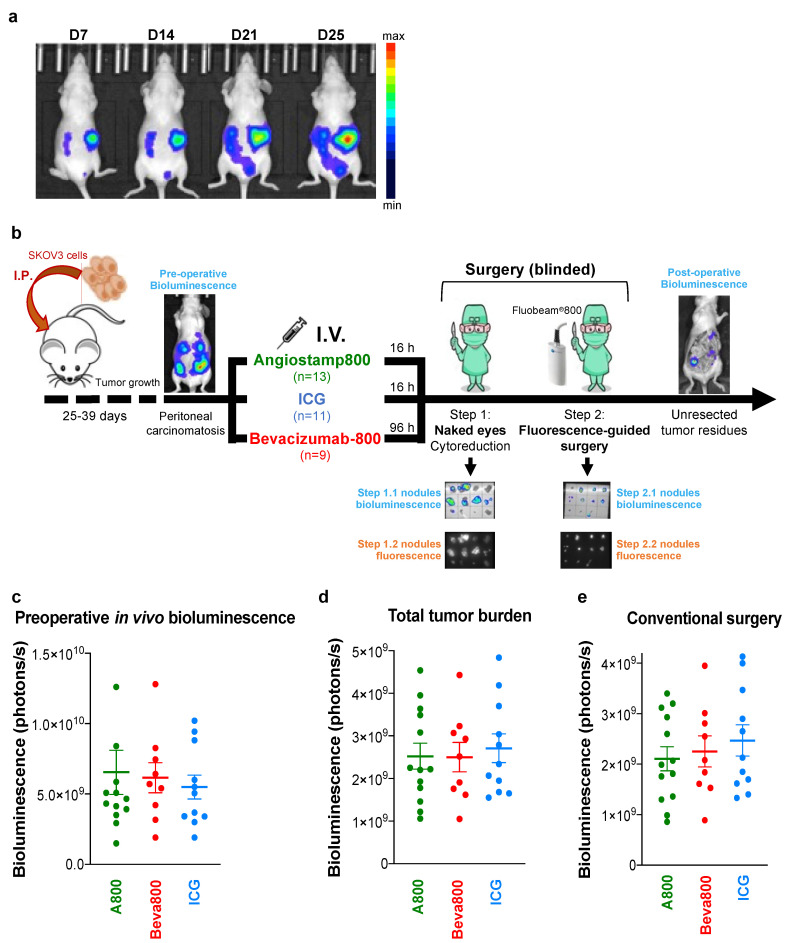
Conventional surgery for peritoneal carcinomatosis. (**a**): Orthotopic murine model of peritoneal carcinomatosis from ovarian cancer. Mice were inoculated with SKOV3-Luc cells intraperitoneally. Tumor growth and dissemination of tumor cells in the peritoneal cavity were followed by in vivo bioluminescence imaging over time. (**b**): Schematic representation of orthotopic peritoneal carcinomatosis murine model, blinded surgery plan, and surgical nodules analysis. Tumor loads resected at each step and the unresected tumor residues left in the mouse body were evaluated by ex vivo bioluminescence imaging of the surgical specimens and the mouse abdominal cavity, respectively. (**c**): In vivo noninvasive bioluminescence in mice with established orthotopic peritoneal carcinomatosis from ovarian cancer showed no significant difference between the groups. (**d**): The total tumor burden is the sum of ex vivo bioluminescence signals of tumor nodules resected by conventional and fluorescence-guided surgery and residual tumor nodules left in mice. No significant differences were observed between the groups. (**e**): Bioluminescence signal of tumor nodules resected by conventional surgery in mice showed no significant difference between the groups. (**d**,**e**): Each dot represents one mouse. Bars, mean ± SEM.

We analyzed whether fluorescence imaging improved surgery in mice with established peritoneal carcinomatosis from ovarian cancer (Figure 1a,b). Mice were distributed into three comparable groups of mice based on noninvasive in vivo bioluminescence imaging evaluating preoperative tumor burden (Figure 1c). Total tumor burden evaluated after surgery also showed no significant differences between groups (Figure 1d).

**Figure 2:**
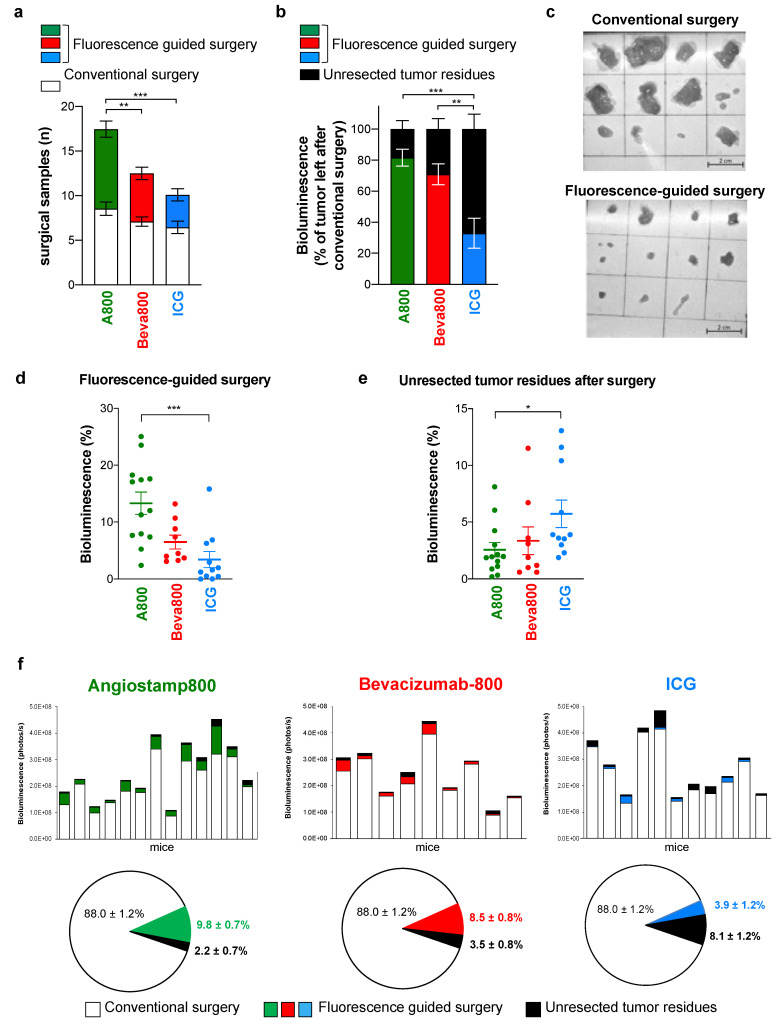
Angiostamp800 and Bevacizumab-IRDye 800CW improved fluorescence-guided surgery for peritoneal carcinomatosis. (**a**): Mean number of tumor nodules resected by conventional and fluorescence-guided surgery. ** *p* = 0.004; *** *p* < 0.001; two-way ANOVA with Tukey post-hoc tests. (**b**): Bioluminescence of tumor resected by fluorescence-guided surgery and of unresected tumor residues expressed as a percentage of tumor left after conventional surgery (mean ± SEM). ** *p* = 0.003; *** *p* < 0.001; two-way ANOVA with Tukey post-hoc tests. (**c**): Representative images of nodules resected by conventional and fluorescence-guided surgery. Scale bar = 2 cm. (**d**): Bioluminescence signals of tumor nodules resected by fluorescence-guided surgery expressed as the percentage of total tumor burden in mice. *** *p* < 0.001; Kruskal–Wallis test with Dunn’s multiple comparisons post-hoc tests. (**e**): Bioluminescence signals of unresected tumor residues after the 2-steps surgery, expressed as the percentage of total tumor burden in mice. * *p* = 0.04; Kruskal–Wallis test with Dunn’s multiple comparison post-hoc tests. (**d**,**e**): Each dot represents one mouse. Bars, mean ± SEM. (**f**): Histograms show bioluminescence signals of tumor nodules resected by conventional surgery, fluorescence-guided surgery, and unresected tumor residues in each mouse. Pies show bioluminescence of tumor nodules resected by conventional surgery, fluorescence-guided surgery, and unresected tumor residues (false-negative fluorescent signal) in every mouse and expressed as the percentage of total tumor burden (mean ± SEM).

The standard conventional surgical procedure without fluorescent-guided surgery, performed by the naked eye and under white light, removed 88.0% ± 1.2 of the total tumor burden without a significant difference in the number and bioluminescence signal of resected nodules between the three groups (Figure 1e and Figure 2a). Fluorescence-guided surgery was then performed without the surgeon being informed of the probes used (blinded fluorescence-guided surgery) and allowed the resection of further tumor nodules invisible to the naked eye (Figure 2a,b), some of them being infra-millimetric (Figure 2c). Fluorescence-guided surgery with Angiostamp800 and Bevacizumab-IRDye 800CW was compared to fluorescence-guided surgery with ICG. More nodules were resected with Angiostamp800 than with Bevacizumab-IRDye 800CW or ICG. Furthermore, the percentage of tumor resected by fluorescence-guided surgery was significantly higher with Angiostamp800 and Bevacizumab-IRDye 800CW than with ICG (Figure 2b,d). Consequently, the amount of unresected tumor residues in mice was significantly lower with Angiostamp800 or Bevacizumab-IRDye 800CW than with ICG (Figure 2e,f). The tumor resection thus corresponded to 97.8% ± 0.7 and 96.5% ± 0.8 of total tumor burden when conventional surgery was completed by fluorescence-guided surgery with Angiostamp800 or Bevacizumab-IRDye 800CW, respectively, and to 91.9% ± 1.2 with ICG (Figure 2f).

### 3.4. In Contrast to ICG, Angiostamp800 and Bevacizumab-IRDye 800CW Further Improved Surgery for Peritoneal Carcinomatosis after Neoadjuvant Chemotherapy

**Figure 3:**
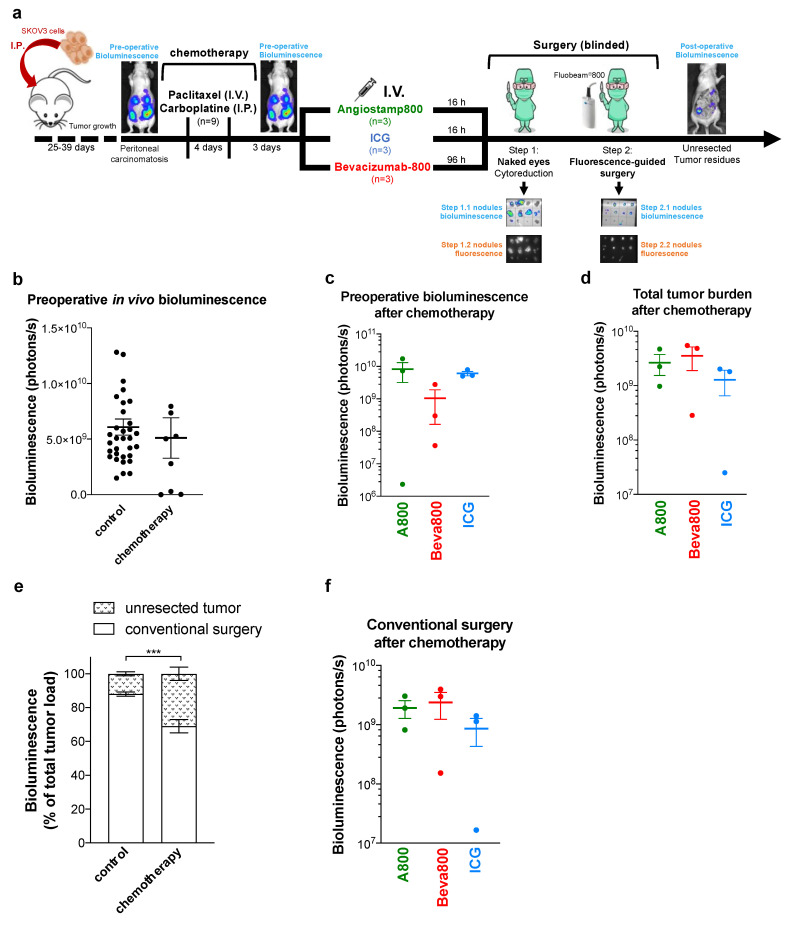
Neoadjuvant chemotherapy decreased peritoneal carcinomatosis. (**a**): Schematic representation of orthotopic peritoneal carcinomatosis growth, treatment, and blinded surgery plan. Mice with well-established peritoneal carcinomatosis were treated with two doses of chemotherapy (paclitaxel 10 mg/kg i.v. + carboplatin 40 mg/kg i.p.) at 4-day intervals. Three days later, mice were imaged with in vivo bioluminescence imaging and distributed into three groups of mice treated by intravenous (I.V.) administration of Angiostamp800 (*n* = 3), or ICG (*n* = 3) for 16 h, or Bevacizumab-IRDye 800CW (*n* = 3) for 96 h. Conventional surgery was first performed, followed by NIR-fluorescence-guided surgery. The mouse body was finally exposed to bioluminescence imaging. The bioluminescence and fluorescence of all surgical samples were measured ex vivo. (**b**): In vivo noninvasive bioluminescence imaging 3 days after chemotherapy in non-treated and chemotherapy-treated mice. (**c**): In vivo preoperative bioluminescence imaging 3 days after chemotherapy showed no significant difference between the groups. (**d**): Total tumor burden after chemotherapy showed the sum of ex vivo bioluminescence of tumor nodules resected by conventional and fluorescence-guided surgery and tumor residues left in mouse body, without significant difference between the groups. (**e**): Bioluminescence of tumor resected by conventional surgery and of unresected tumor residues expressed as a percentage of total tumor load in mice operated with (*n* = 9) or without (control, *n* = 33) neoadjuvant chemotherapy (mean ± SEM). *** *p* < 0.001; two-way ANOVA with Sidak post-hoc tests. (**f**): Bioluminescence signal of tumor nodules resected by conventional surgery after neoadjuvant chemotherapy in mice showed no significant difference between the groups. (**b**–**d**,**f**): Each dot represents one mouse. Bars, mean ± SEM.

In order to simulate the clinical situation as closely as possible and evaluate the effects of chemotherapy on probe specificity in relation to inflammation or tissue remodeling, we further compared the three probes for fluorescence-guided surgery in mice with peritoneal carcinomatosis after neoadjuvant chemotherapy (Figure 3a).

**Figure 4:**
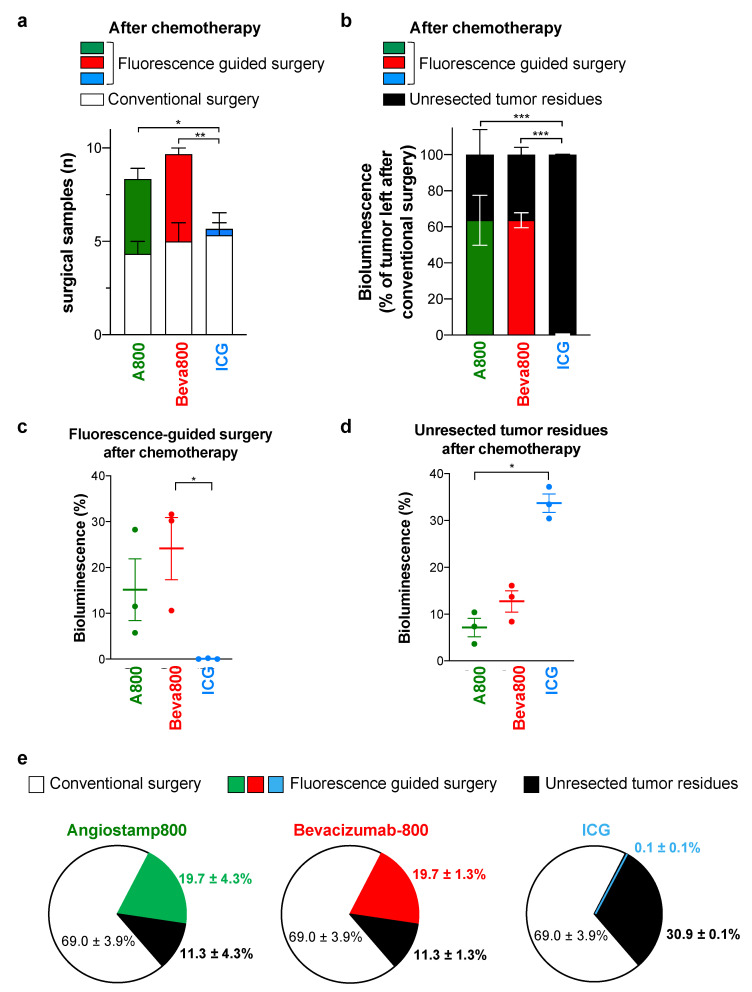
Angiostamp800 and Bevacizumab-IRDye 800CW, but not ICG, improved fluorescence-guided surgery for peritoneal carcinomatosis after chemotherapy. (**a**): Mean number of tumor nodules resected by conventional and fluorescence-guided surgery after neoadjuvant chemotherapy. * *p* = 0.01; ** *p* = 0.004; two-way ANOVA with Tukey post-hoc tests. (**b**): Bioluminescence of tumor resected by fluorescence-guided surgery and unresected tumor residues left in mouse body, expressed as a percentage of tumor left after conventional surgery in mice operated after neoadjuvant chemotherapy (mean ± SEM). *** *p* < 0.001; two-way ANOVA with Tukey post-hoc tests. (**c**): Bioluminescence signals of tumor nodules resected by fluorescence-guided surgery in mice operated after chemotherapy and expressed as the percentage of total tumor burden in mice. * *p* = 0.02; Kruskal–Wallis test with Dunn’s multiple comparison post-hoc tests. (**d**): Bioluminescence signals of unresected tumor residues after the 2-step surgery in mice after chemotherapy, and expressed as the percentage of total tumor burden in mice. * *p* = 0.01; Kruskal–Wallis test with Dunn’s multiple comparison post-hoc tests. (**c**,**d**): Each dot represents one mouse. Bars, mean ± SEM. (**e**): Pies show bioluminescence of tumor nodules resected by conventional and fluorescence-guided surgery and of unresected tumor residues (false-negative fluorescent signal) in mice operated on after chemotherapy and expressed as the percentage of total tumor burden (mean ± SEM).

Mice received two doses of a combination of paclitaxel and carboplatin, reducing the total tumor burden by 16% ± 30 after 3 days (Figure 3b). Mice were distributed into three comparable groups of mice based on preoperative noninvasive in vivo bioluminescence imaging evaluating initial tumor burden after chemotherapy (Figure 3c). Total tumor burden evaluated after surgery also showed no significant differences between groups (Figure 3d). Tumors removed by conventional surgery after chemotherapy corresponded to 69.0% ± 3.9 of the total tumor burden, which was lower than tumor nodules removed by conventional surgery without prior chemotherapy (Figure 3e). This resulted from the reduction of the tumor burden by chemotherapy, leading to fewer and smaller tumor nodules that were more difficult to see with the naked eye. No significant difference was observed between the groups after conventional surgery (Figure 3f), but blinded fluorescence-guided surgery with Angiostamp800 and Bevacizumab-IRDye 800CW allowed the removal of additional tumor nodules (Figure 4a–c). In contrast, a blurred fluorescence signal with no tumor contrast was observed throughout the peritoneal cavity with ICG (Supplementary Figure S5), therefore limiting fluorescence-guided tumor resection (Figure 4a–d). Consequently, after neo-adjuvant chemotherapy, the 2-step tumor resection removed 88.7% ± 4.3 and 88.7% ± 1.3 of the total tumor burden when conventional surgery was completed by fluorescence-guided surgery with Angiostamp800 or Bevacizumab-IRDye 800CW, respectively (Figure 4e). In contrast, ICG did not improve surgery at all.

### 3.5. Angiostamp800 and Bevacizumab-IRDye 800CW Showed Higher Specificity and Lower False-Positive Rate than ICG

Angiostamp800 and Bevacizumab-IRDye 800CW showed higher specific tumor uptake and detection of tumor nodules by fluorescence signal than ICG. When using intraoperative fluorescence imaging in parallel with bioluminescence imaging to reveal the presence of peritoneal carcinomatosis in mice, we observed a colocation of Angiostamp800 and Bevacizumab-IRDye 800CW signals with most bioluminescence-positive nodules. In contrast, ICG failed to detect several bioluminescent tumor nodules (false-negative fluorescence signal) (Supplementary Figure S5). Moreover, ICG fluorescence signal was observed in more tumor-free tissues (false-positive fluorescence signal) than Angiostamp800 and Bevacizumab-IRDye 800CW. In addition, as already observed in the biodistribution study, some fluorescence signal was also observed in kidneys and uterus with Angiostamp800, liver, and uterus with Bevacizumab-IRDye 800CW, and liver, uterus, and kidneys with ICG.

**Figure 5:**
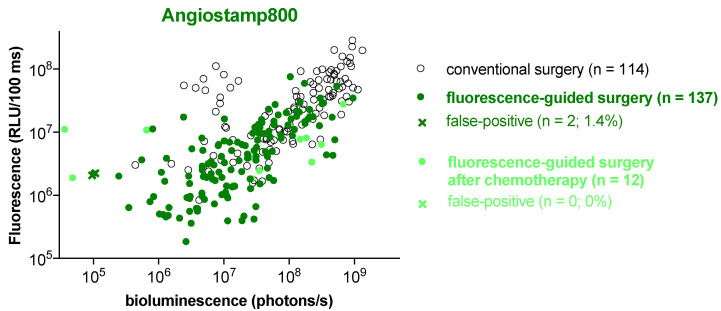

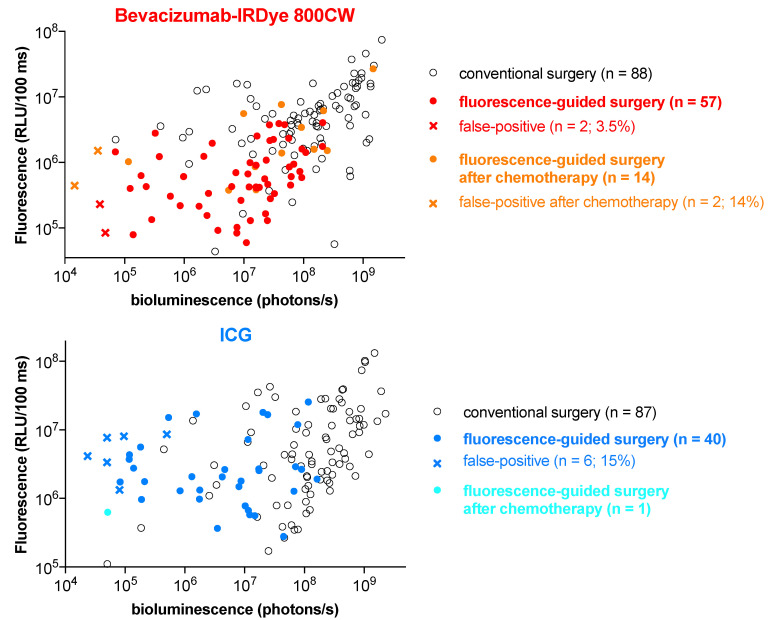
Angiostamp800 and Bevacizumab-IRDye 800CW showed fewer false-positive fluorescent signals than ICG. All fluorescent surgical nodules from mice with peritoneal carcinomatosis without or after chemotherapy were analyzed ex vivo by bioluminescence to determine their tumor status. Nodules with low or no bioluminescence signals were further analyzed to quantify luciferase expression by measuring the presence of tumor cells. Data show a correlation between the bioluminescence and fluorescence of samples resected by conventional surgery (open black dots) or fluorescence-guided surgery (colored plain dots). Fluorescent nodules with a negative bioluminescence signal were considered false positives (colored crosses). The number of resected nodules and the percentage of false-positive residues is indicated for each probe, except for fluorescence-guided surgery with ICG after chemotherapy, which failed to resect additional tumor nodules because of the blurred fluorescence signal from ICG.

An exhaustive comparison of ex vivo bioluminescence and fluorescence signals of all surgical specimens confirmed that Angiostamp800 and Bevacizumab-IRDye 800CW provided less non-specific signals than ICG (Figure 5). The fluorescence signals from specimens removed by conventional surgery strongly correlated with their bioluminescence signals (Spearman’s correlation coefficient *r* = 0.645, *p* < 0.001 for Angiostamp800; *r* = 0.467, *p* < 0.001 for Bevacizumab-IRDye 800CW; *r* = 0.477, *p* < 0.001 for ICG) (Figure 5), indicating proportionality between tissue fluorescence labeling and tumor burden. In addition, specimens removed by fluorescence-guided surgery had lower bioluminescence signals than those removed by conventional surgery, confirming that they were smaller. An even stronger correlation between fluorescence and bioluminescence signals was observed in specimens collected by fluorescence-guided surgery with Angiostamp800 (Spearman’s correlation coefficient *r* = 0.710, *p* < 0.001) and Bevacizumab-IRDye 800CW (Spearman’s correlation coefficient *r* = 0.443, *p* < 0.001), but not with ICG, which did not lead to the resection of additional tumor nodules. Interestingly, although Angiostamp800 detected many additional tumor nodules without false-positive fluorescence signals after neoadjuvant chemotherapy, only Bevacizumab-IRDye 800CW showed a correlation between fluorescence and bioluminescence signals (Spearman’s correlation coefficient *r* = 0.626, *p* = 0.02). ICG showed more false-positive fluorescence signals than Angiostamp800 and Bevacizumab-IRDye 800CW (Figure 5).

## 4. Discussion

Complete surgical removal of lesions is the most important factor in curing and preventing recurrence in peritoneal carcinomatosis. Intraoperative fluorescence imaging has been developed to help surgeons quickly identify and localize very small or invisible tumor nodules. In this study, we compare for the first time to our knowledge the tumor-specific molecular probes Bevacizumab-IRDye 800CW and Angiostamp800 to the FDA- and EMA-approved non-targeted fluorescent agent ICG for fluorescence-guided surgery of peritoneal carcinomatosis of ovarian origin. We show that all three probes accumulate in ovarian tumors and peritoneal metastases, but Angiostamp800 and Bevacizumab-IRDye 800CW show more specific tumor uptake and tumor nodule detection than ICG. In addition, Bevacizumab-IRDye 800CW and Angiostamp800, in contrast to ICG, improve peritoneal carcinomatosis surgery in the first-line and after neoadjuvant chemotherapy, with a low false-positive rate and a low residual tumor burden.

The molecular probes used in this study are radically different in nature: ICG is a small, natively fluorescent organic molecule that has long been known to passively accumulate in cancerous tissues through the EPR effect [5,6,7,8,9,10]. Bevacizumab is a monoclonal antibody specifically directed against VEGF, which is already widely used in the clinic as targeted therapy and has recently been labeled with the IRDye 800CW fluorophore for fluorescence-guided surgery applications [5,11,12,13,15]. Angiostamp800 is a multimeric cyclopeptide template that has been specifically designed to target α_v_β_3_ integrin with high affinity and specificity and has been conjugated to a NIR fluorescent dye for in vivo imaging of α_v_β_3_ integrin overexpressing cells, such as endothelial cells during tumor neoangiogenesis and aggressive migrating tumor cells during metastasis [16,17,18,23,24]. As a direct consequence of their respective chemical nature, the in vivo pharmacokinetics of these three probes are different. Furthermore, the fluorophore choice is known to influence tumor-specific fluorescent signal, biodistribution, and clearance [34,35]. Although IRDye 800CW is the most commonly used NIR fluorophore in clinical settings [3,5], it has shown non-specific fluorescence accumulation [35]. Other fluorophores, such as zwitte-rionic NIR fluorophore-based probes, have shown more favorable in vivo imaging characteristics [34,35] and should be investigated. Consequently, the chemical nature of the molecular probes and the conjugated fluorophore will determine the biodistribution of the probes and the optimal time to perform surgery for each. Studies using ICG at different time points, ranging from 30 min to 72 h for fluorescence imaging after ICG injection, have been reported in peritoneal carcinomatosis [9,10,36,37]. Based on our in vivo pharmacokinetics and biodistribution studies in mice with subcutaneous tumors, we determined that the optimal time to perform surgery is between 16 and 24 h after injection for ICG and Angiostamp800, as previously shown [19,24]. In contrast, Bevacizumab-IRDye 800CW requires several days to reach a high tumor signal with a stabilized tumor-to-healthy tissue ratio, consistent with previous studies [12,13,15,32,33,38]. It was injected 96 h before surgery.

Fluorescent probes for cancer imaging based on active targeting of specific tumor biomarkers are expected to provide better contrast between tumor and healthy tissues than non-targeted agents that passively accumulate in the tumor via the EPR effect [5]. Various cancer biomarkers associated with ovarian cancers have been considered for the design of tumor-targeting agents, including the folate receptor, VEGF, EGFR, HER2, epCAM, PSMA, and α_v_β_3_ integrin [3,5]. From our data and the literature, ICG [8,9,10,36,37], Angiostamp800 [22,23,24], and Bevacizumab-IRDye-800CW [15,33,39] accumulate in subcutaneous ovarian tumors, as well as in peritoneal small tumor nodules after intravenous injection, demonstrating their interest in tumor localization. The specificity of Bevacizumab-IRDye 800CW and Angiostamp800 for their respective targets VEGF and α_v_β_3_ integrin has been widely demonstrated [3,11,12,13,14,15,16,17,18,19,20,21,22,23,24,33]. Bevacizumab-IRDye 800CW and Angiostamp800 also show significant signals in the uterus and ovaries in the absence of tumor cells, which could be explained by their high level of angiogenesis expressing VEGF and α_v_β_3_ integrin during the phases of endometrial cycle in mammals [40].

Intraoperative image-guided tumor location can increase the effectiveness of surgery for treating peritoneal metastases. Accordingly, Angiostamp800, Bevacizumab-IRDye-800CW, and to a lower extent ICG, improve first-line surgery by detecting specifically human ovarian tumor nodules that are undetectable without fluorescence imaging. We observe non-specific fluorescence of ICG in mice with peritoneal micrometastases, as already reported [8,9,10,36]. In addition, about 8% of the total tumor load is not detected with ICG (false-negative fluorescent signal), leading to incomplete tumor resection. In contrast, when using Angiostamp800 and Bevacizumab-IRDye 800CW, almost all tumor nodules are resected, with only 2.2% and 3.5% undetected tumor burden (false-negative fluorescent signals), respectively, when cytoreductive surgery is completed by fluorescence-guided surgery. In agreement, the high potential of Bevacizumab-IRDye 800CW for fluorescence-guided surgery has already been identified, resulting in its current use in several clinical trials for cancer surgery [3,11,12,13]. In particular, Bevacizumab-IRDye 800CW is safe and can be used to treat peritoneal carcinomatosis from colon origin [15,33], whose etiology is very similar to that of ovarian origin. NIR optical imaging-guided surgery using Angiostamp800 also improves the quality of tumor and metastases resection, including in ovarian cancer and peritoneal carcinomatosis in preclinical models [3,22,23,24,41].

In some cases of peritoneal carcinomatosis, neoadjuvant platinum-based chemotherapy is indicated to decrease the tumor load before debulking surgery. Here, we show that the rate of tumor resection by conventional surgery decreases in mice submitted to neoadjuvant chemotherapy, indicating that the smaller the tumor nodules, the more difficult the surgery. Despite the small number of animals studied after neoadjuvant chemotherapy, our data show that Angiostamp800 and Bevacizumab-IRDye 800CW have value in improving tumor resection when used after neoadjuvant chemotherapy. Conversely, ICG shows a blurry fluorescent signal, probably related to inflammation, tissue remodeling, and fibrosis of the peritoneum caused by chemotherapy [42]. As a result, ICG compromises tumor identification, resulting in a residual tumor burden of 31% (false-negative fluorescent signal) when cytoreductive surgery and fluorescence-guided surgery are performed after neoadjuvant chemotherapy. In contrast, both Angiostamp800 and Bevacizumab-IRDye 800CW remove >90% of the total tumor burden.

In addition to achieving high sensitivity by detecting all tumor nodules, the challenge of intraoperative fluorescence imaging is also to provide a low false-positive rate so as not to increase surgical aggressiveness. Bioluminescence imaging, which is highly sensitive (a few tens of cells can be detected), allows us to assess the tumor status of surgical nodules. By analyzing luciferase expression in all specimens with a bioluminescence signal below the detection limit, we avoid misinterpretation for tumor specimens that would have lost their bioluminescence signal due to prolonged surgery or lack of oxygen after excision. The strong correlation between bioluminescence signals from the surgical nodules and fluorescence signals from Angiostamp800 and Bevacizumab-IRDye 800CW demonstrate their high tumor-targeting specificity. Accordingly, Angiostamp800 and Bevacizumab-IRDye 800CW fluorescence-guided surgery led to the resection of a few false-positive specimens (1.4% and 3.5%, respectively) compared to ICG (15%) in first-line surgery. The higher number of false-positive surgical nodules collected when using ICG can be interpreted as a lack of specificity of the enhanced permeability and retention effect.

Interestingly, after neoadjuvant chemotherapy, Angiostamp800 leads to no false-positive surgical specimens, while Bevacizumab-IRDye 800CW false-positive rate rises up to 14%. ICG shows a non-specific signal throughout, which precludes any fluorescence-guided resection. In agreement, it has been previously reported that chemotherapy prior to fluorescence-guided surgery with ICG or Bevacizumab-IRDye 800CW increases the false-positive detection rate due to inflammation-induced hyperplasia [15,42].

## 5. Conclusions

In conclusion, complete tumor resection has the greatest impact on survival and relapse for patients with peritoneal carcinomatosis. Comparing, for the first-time, fluorescence-guided surgery in ovarian peritoneal carcinomatosis using non-specific or targeted molecular probes, we show that the targeted probes Angiostamp800 and Bevacizumab-IRDye 800CW provide better detection of tumors and metastases than the clinically used fluorescent tracer ICG. Tumor-specific targeted probes help surgeons identify and completely remove tumors while limiting aggressiveness to surrounding normal tissues. Targeted contrast agents, such as Bevacizumab-IRDye 800CW or possibly Angiostamp800, could be an appropriate alternative to ICG in the clinic, including after neoadjuvant chemotherapy.

## Data Availability

Data are available upon request.

## Supplementary Materials

The following supporting information can be downloaded at: https://www.mdpi.com/article/10.3390/biomedicines10051059/s1, Figure S1: Angiostamp800, Bevacizumab-IRDye 800CW, and ICG fluorescence detectability; Figure S2: Bevacizumab-IRDye 800CW had longer circulation time in the blood than Angiostamp800 and ICG; Figure S3: Angiostamp800, Bevacizumab-IRDye 800CW, and ICG accumulated in subcutaneous SKOV3 tumors; Figure S4: Angiostamp800, Bevacizumab-IRDye 800CW, and ICG accumulated in subcutaneous SKOV3 tumors ex vivo; Figure S5: ICG showed more false-positive signal than Angiostamp800 and Bevacizumab-IRDye 800CW; Table S1: Molecular probes and device.
