# Supplementary material for: Tumor-Specific Imaging with Angiostamp800 or Bevacizumab-IRDye 800CW Improves Fluorescence-Guided Surgery over Indocyanine Green in Peritoneal Carcinomatosis
Source: Biomedicines. 2022 May 3;10(5):1059. doi: 10.3390/biomedicines10051059 (PMC9138305; doi:10.3390/biomedicines10051059)
Supplement: Supplementary file 1 [file biomedicines-10-01059-s001.zip › Supplementary_Figure_S1.pdf]

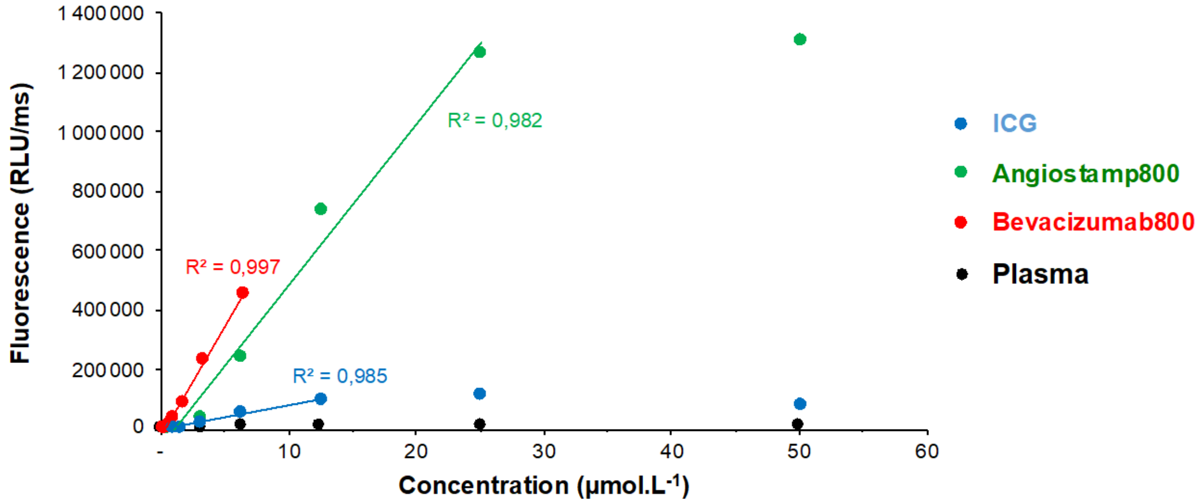

Figure S1 Angiostamp800, Bevacizumab-IRDye 800CW, and ICG fluorescence detectability. Fluorescence signals of 10 µL of decreasing concentrations of Angiostamp800, Bevacizumab-IRDye 800CW and ICG diluted in murine plasma. The highest concentrations of Angiostamp800 and ICG underwent fluorescence quenching. Fluorescence detection was linear for probe concentrations that provided significantly higher specific signals than background (14 $nmol.L^{-1}$ for Bevacizumab-IRDye 800CW, 46 $nmol.L^{-1}$ for Angiostamp800 and 170 $nmol.L^{-1}$ for ICG).
