# Supplementary material for: Tumor-Specific Imaging with Angiostamp800 or Bevacizumab-IRDye 800CW Improves Fluorescence-Guided Surgery over Indocyanine Green in Peritoneal Carcinomatosis
Source: Biomedicines. 2022 May 3;10(5):1059. doi: 10.3390/biomedicines10051059 (PMC9138305; doi:10.3390/biomedicines10051059)
Supplement: Supplementary file 1 [file biomedicines-10-01059-s001.zip › Supplementary_Figure_S2.pdf]

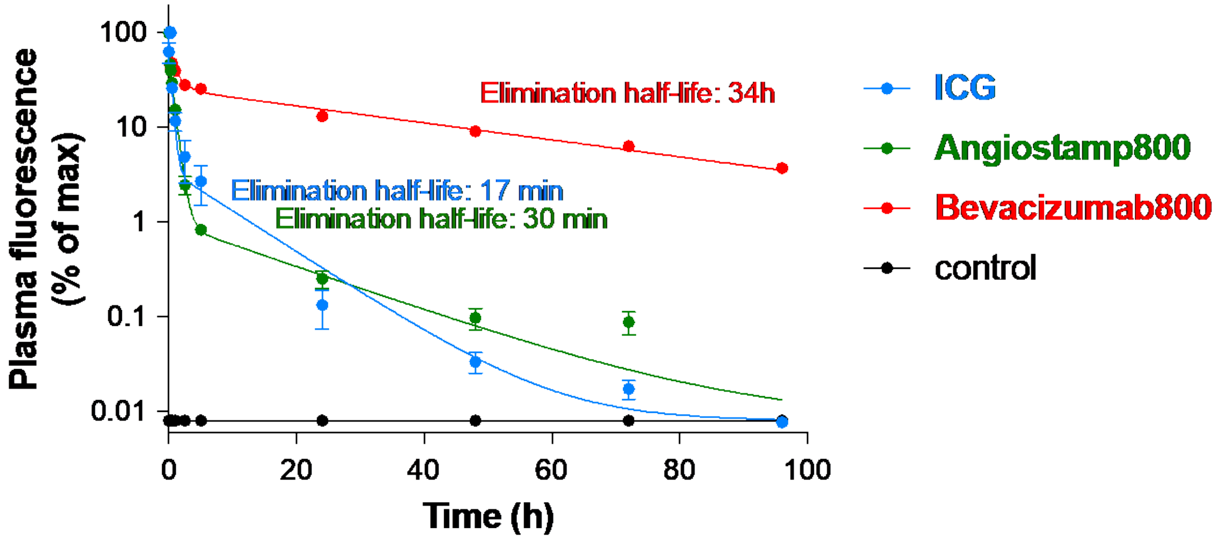

Figure S2 Bevacizumab-IRDye 800CW had longer circulation time in the blood than Angiostamp800 and ICG. Healthy mice were injected intravenously with Angiostamp800, Bevacizumab-IRDye 800CW, or ICG, and fluorescence imaging of blood plasma samples collected at different time points were performed. Results are expressed as a percentage of the initial maximum signal (mean ± SEM; n = 3).
