# Supplementary material for: Tumor-Specific Imaging with Angiostamp800 or Bevacizumab-IRDye 800CW Improves Fluorescence-Guided Surgery over Indocyanine Green in Peritoneal Carcinomatosis
Source: Biomedicines. 2022 May 3;10(5):1059. doi: 10.3390/biomedicines10051059 (PMC9138305; doi:10.3390/biomedicines10051059)
Supplement: Supplementary file 1 [file biomedicines-10-01059-s001.zip › Supplementary_Figure_S3.pdf]

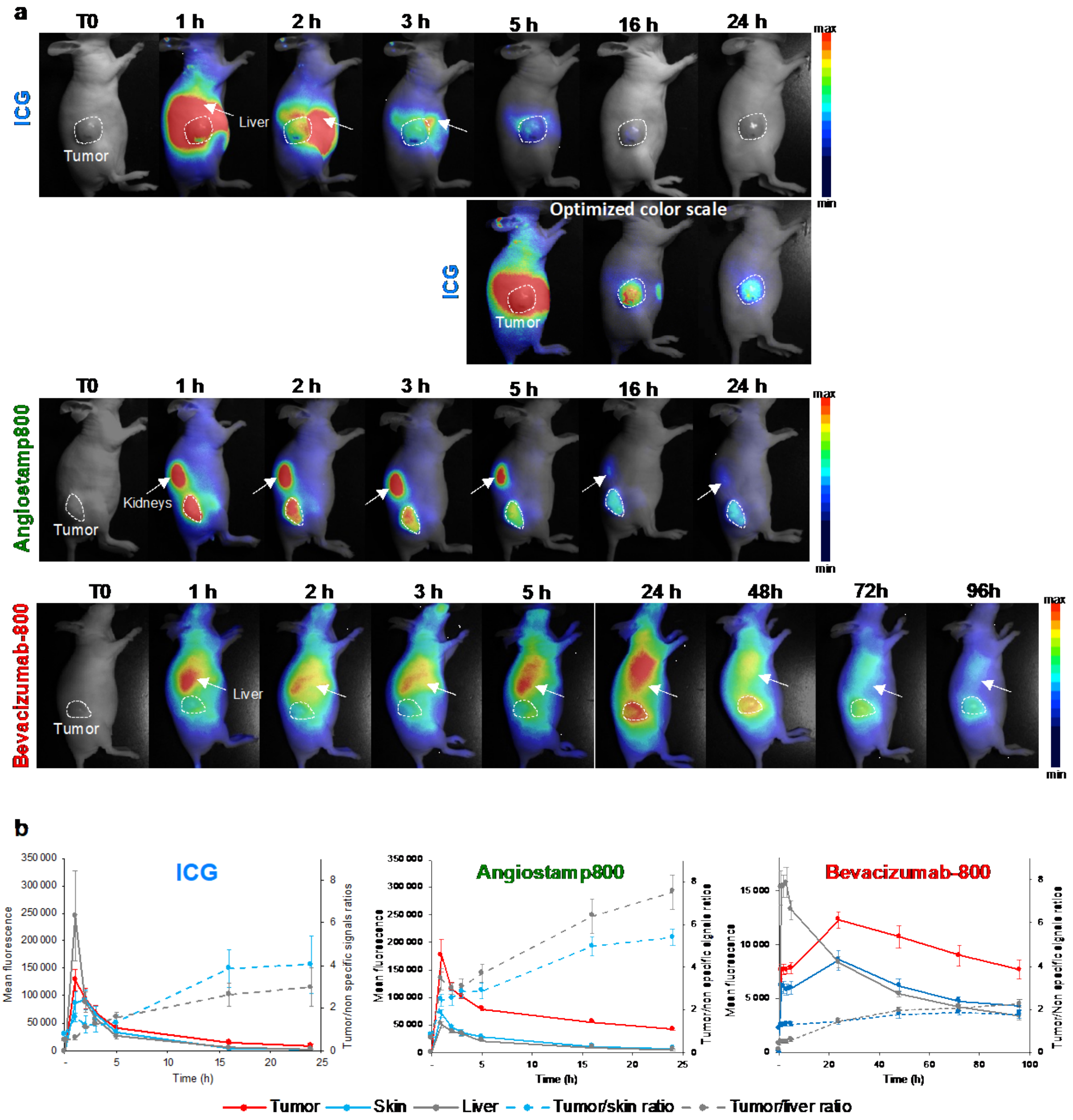

Figure S3 Angiostamp800, Bevacizumab-IRDye 800CW, and ICG accumulated in subcutaneous SKOV3 tumors. Mice with SKOV3 subcutaneous tumors were injected intravenously with Angiostamp800, Bevacizumab-IRDye 800CW, or ICG. **a:** Representative fluorescence images at different times after injection are shown (Angiostamp800 and ICG: 10 ms integration time, min-max: 2121-13200, and optimized color scale min-max: 279-2607; Bevacizumab IRDye 800CW: 200 ms integration time, min-max: 3006-29710). Dotted lines show subcutaneous tumors, arrows show kidneys or liver as indicated. **b:** Regions of interest (ROIs) are defined on tumor, liver, and skin to semi-quantify the amount of photons detected per pixel. The results are expressed as the mean fluorescence (RLU/pixel/100 ms) ± SEM in tumor, skin, and liver, and as the mean tumor-to-skin and tumor-to-liver fluorescence ratios ± SEM (Angiostamp800 and ICG, n = 4; Bevacizumab-IRDye 800CW, n = 6).
