# Supplementary material for: Tumor-Specific Imaging with Angiostamp800 or Bevacizumab-IRDye 800CW Improves Fluorescence-Guided Surgery over Indocyanine Green in Peritoneal Carcinomatosis
Source: Biomedicines. 2022 May 3;10(5):1059. doi: 10.3390/biomedicines10051059 (PMC9138305; doi:10.3390/biomedicines10051059)
Supplement: Supplementary file 1 [file biomedicines-10-01059-s001.zip › Supplementary_Figure_S4.pdf]

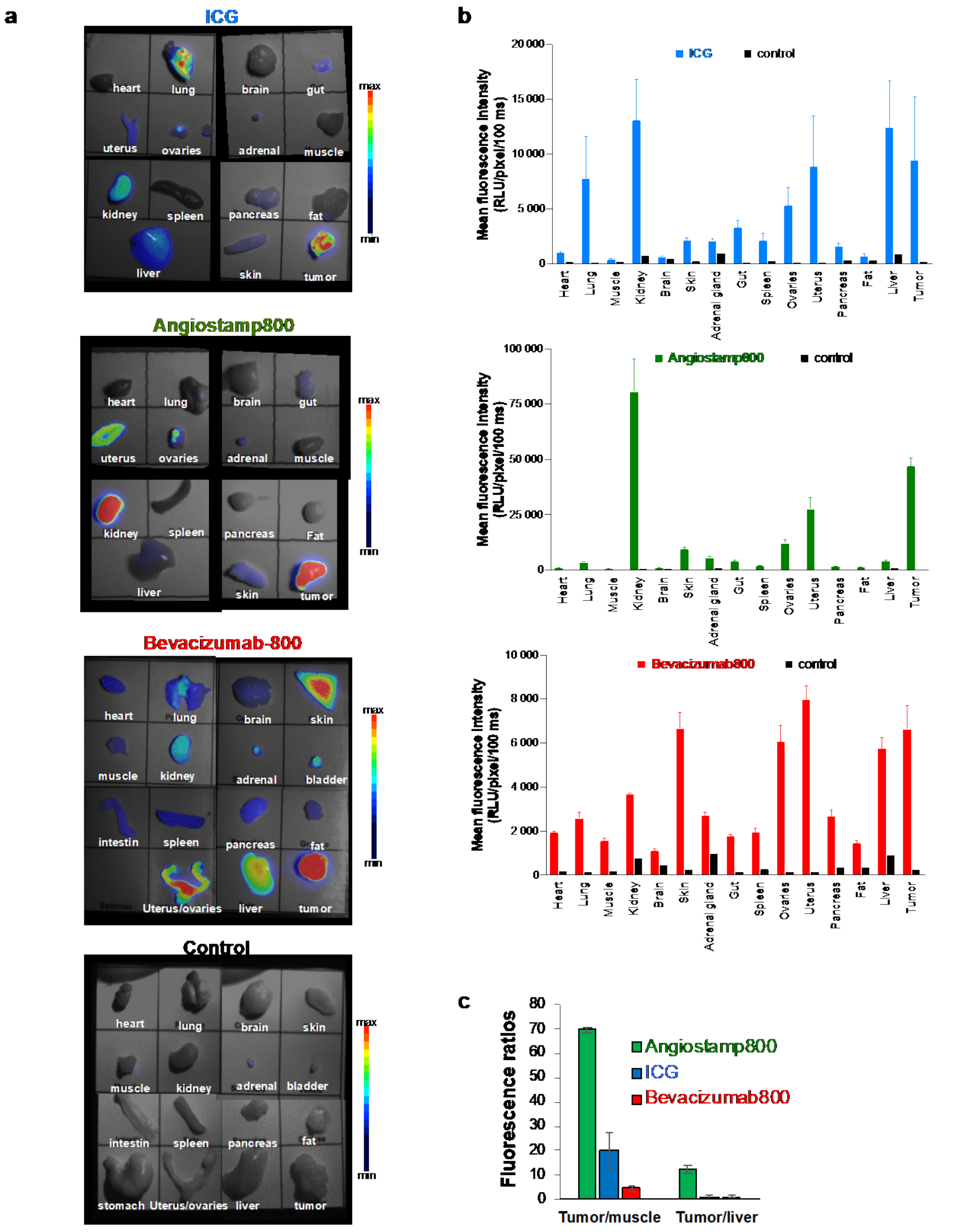

Figure S4 Angiostamp800, Bevacizumab-IRDye 800CW, and ICG accumulated in subcutaneous SKOV3 tumors *ex vivo*. **a:** Representative fluorescence images of isolated organs collected 24 h after intravenous injection of Angiostamp800 and ICG (50 ms integration time, min-max: 2028-25931), and

in non-injected mouse (control), or 96 h after intravenous injection of Bevacizumab-IRDye 800CW (500 ms integration time, min-max: 4007-39839). **b:** Fluorescence images were performed on isolated organs. ROIs are defined on the extracted organs to semi-quantify the amount of photons detected per pixel. The results are expressed as the mean (RLU/pixel/100 ms) ± SEM in SKOV3 tumor-bearing mice (n = 3) and non-injected mice (control). **c:** ROIs were defined on tumor, liver, and muscle to semi-quantify the amount of photons detected per pixel. The results are expressed as the mean tumor-to-muscle and tumor-to-liver fluorescence ratios ± SEM (n=6) at 24 h (Angiostamp800 and ICG, n = 4), or 96 h (Bevacizumab-IRDye 800CW, n = 6).
