# Supplementary material for: Tumor-Specific Imaging with Angiostamp800 or Bevacizumab-IRDye 800CW Improves Fluorescence-Guided Surgery over Indocyanine Green in Peritoneal Carcinomatosis
Source: Biomedicines. 2022 May 3;10(5):1059. doi: 10.3390/biomedicines10051059 (PMC9138305; doi:10.3390/biomedicines10051059)
Supplement: Supplementary file 1 [file biomedicines-10-01059-s001.zip › Supplementary_Figure_S5.pdf]

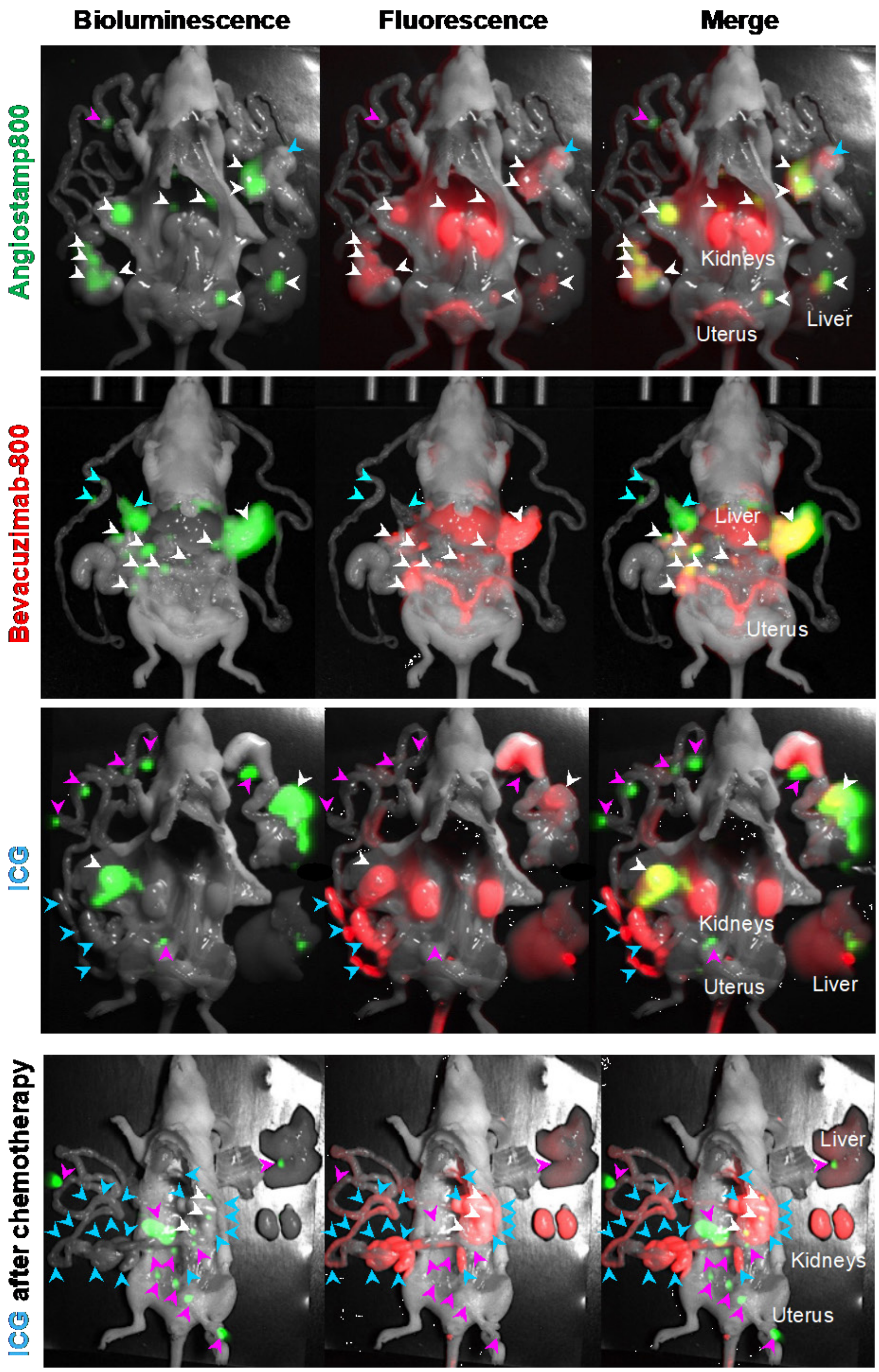

Figure S5 ICG showed more false-positive signal than Angiostamp800 and Bevacizumab-IRDye 800CW. Mice with established peritoneal carcinomatosis, treated with or without prior neoadjuvant chemotherapy, were injected intravenously with Angiostamp800, or ICG for 16 h, or Bevacizumab-IRDye 800CW for 96 h. Mice were submitted to intraoperative fluorescence in combination with bioluminescence. Representative images of one operated mouse per group, and with extended organs exposition are shown. Bioluminescence showed SKOV3 cells in the peritoneal cavity (in green) and 2D-fluorescence imaging showed Angiostamp800, ICG, or Bevacizumab-IRDye 800CW location (in red). White arrowheads show fluorescence and SKOV3 tumor nodules signals colocation (in yellow); pink arrowheads show SKOV3 tumor nodules signals without fluorescence signal (false-negative fluorescence signal in green); blue arrowheads show fluorescence signals without bioluminescence signal (false-positive fluorescence signal in red). In mice operated after chemotherapy, ICG provided a blurry signal (in red) all over the peritoneal cavity with no longer informative contrast.
