# Supplementary material for: Tumor-Specific Imaging with Angiostamp800 or Bevacizumab-IRDye 800CW Improves Fluorescence-Guided Surgery over Indocyanine Green in Peritoneal Carcinomatosis
Source: Biomedicines. 2022 May 3;10(5):1059. doi: 10.3390/biomedicines10051059 (PMC9138305; doi:10.3390/biomedicines10051059)
Supplement: Supplementary file 1 [file biomedicines-10-01059-s001.zip › Supplementary_Methods.pdf]

Supplementary methods

SKOV3-Luc cells

SKOV3-Luc cells stably expressing Luc2 firefly luciferase were derived from SKOV3 cells. Briefly, lentiviral vectors coding for Luc-IRES-Neo cassette were derived from HIV and have the capacity to transfer genes into non-dividing cells[1]. They were VSV-G pseudotyped, which confers a very broad cell tropism and an increased stability. To prevent transactivation of genes neighboring the integration site by the retroviral long terminal repeat (LTR), we used self-inactivating constructs (SINs) in which the enhancer/promoter sequences of the U3 region in the 3'LTR are deleted. Constructs also carry a central poly-purine tract, a central termination sequence, and a woodchuck hepatitis virus post-transcriptional regulatory element that significantly enhance transduction efficiency and transgene expression[2]. In the vector, transcription is driven by the SFFV promoter. SKOV3 cells were seeded in 12-well plates. After 24 h, lentiviral vector was added (1.8×10^7 UI/mL; 100 µL/well) in renewed medium for 48 h. Selection of stable transduced cells was performed by adding 600 µg/mL G418 (Invivogen, France) to the culture medium. Monoclonal cell populations were isolated by limiting dilution sub-cloning in 96-well plates. Luciferase expressing clones were identified by addition of 150 $\mu g.mL^{-1}$ of D-luciferin (Promega, France) followed by *in vitro* bioluminescence imaging using the IVIS Kinetic imaging system (PerkinElmer). The stability of luciferase expression was checked over several division cycles.

Subcutaneous ovarian tumor model

Mice were injected subcutaneously in the flank with 10^7 SKOV3 cells in 200 µL 1X PBS. Tumor size was measured three times a week using a caliper, and the tumor volume was calculated as length × $(\text{width})^2$ × 0.4.

Pharmacokinetics studies on blood plasma samples

Fifty microliters of blood were sampled from the tail vein before and at different times after probes administration, centrifuged 5 min at 8,000 g, and 10 µL of plasma were used for fluorescence imaging using the Fluobeam®800, as previously described[3-5]. Half-lives were measured from nonlinear regression fit analyses (two phase decay, GraphPad Software Inc., San Diego, CA, USA).

In vivo *biodistribution of fluorescent probes*

When subcutaneous SKOV3 tumor volume reached 300 ± 30 mm^3, mice were anesthetized, and 2D-fluorescence images were acquired using the Fluobeam®800 at several time points after fluorescent probes administration (n = 6 mice per group). At the end of the experiment, mice were sacrificed, and some organs were collected for *ex vivo* imaging using the Fluobeam®800. Semiquantitative data were obtained using the Wasabi® software (Hamamatsu, Massy, France) by drawing regions of interest (ROIs) on the different organs and fluorescence signals were expressed as the number of relative light units per pixel per unit of exposure time (RLU/pixel/100 ms) and relative to the fluorescence signal in the skin, liver or muscle, as previously described[4-6].

References

1. Tomkowiak M, Ghittoni R, Teixeira M, Blanquier B, Szecsi J, Negre D, et al. Generation of transgenic mice expressing EGFP protein fused to NP68 MHC class I epitope using lentivirus vectors. Genesis. 2013;51(3):193-200.

2. Gonzalez-Murillo A, Lozano ML, Alvarez L, Jacome A, Almarza E, Navarro S, et al. Development of lentiviral vectors with optimized transcriptional activity for the gene therapy of patients with Fanconi anemia. Hum Gene Ther. 2010;21(5):623-30.

3. Gilson P, Couvet M, Vanwonterghem L, Henry M, Vollaire J, Baulin V, et al. The pyrrolopyrimidine colchicine-binding site agent PP-13 reduces the metastatic dissemination of invasive cancer cells in vitro and in vivo. Biochem Pharmacol. 2019;160:1-13.

4. Michy T, Massias T, Bernard C, Vanwonterghem L, Henry M, Guidetti M, et al. Verteporfin-Loaded Lipid Nanoparticles Improve Ovarian Cancer Photodynamic Therapy In Vitro and In Vivo. Cancers (Basel). 2019;11(11):1760.

5. Bouclier C, Simon M, Laconde G, Pellerano M, Diot S, Lantuejoul S, et al. Stapled peptide targeting the CDK4/Cyclin D interface combined with Abemaciclib inhibits KRAS mutant lung cancer growth. Theranostics. 2020;10(5):2008-28.

6. Jeannot V, Gauche C, Mazzaferro S, Couvet M, Vanwonterghem L, Henry M, et al. Anti-tumor efficacy of hyaluronan-based nanoparticles for the co-delivery of drugs in lung cancer. J Controlled Release. 2018;275:117-28.
