# Supplementary material for: Tumor-Specific Imaging with Angiostamp800 or Bevacizumab-IRDye 800CW Improves Fluorescence-Guided Surgery over Indocyanine Green in Peritoneal Carcinomatosis
Source: Biomedicines. 2022 May 3;10(5):1059. doi: 10.3390/biomedicines10051059 (PMC9138305; doi:10.3390/biomedicines10051059)
Supplement: Supplementary file 1 [file biomedicines-10-01059-s001.zip › Supplementary_Table_S1.pdf]

Table S1: Molecular probes and device

	Molecular probes			Device
	**Bevacizumab-IRDye 800CW** SurgVision (Netherlands)	**Angiostamp800** Fluoptics (France)	**Indocyanine Green (ICG)** SERB (France)	**Fluobeam®800** Fluoptics (France)
Absorbance and emission peaks	Abs: 774 nm<br>Em: 789 nm	Abs: 781 nm<br>Em: 794 nm	Abs: 790 nm<br>Em: 830 nm	Excitation: 780 nm laser light<br>Emission: >830 nm
Extinction coefficient x1000 ($cm^{-1}.M^{-1}$)	240	Unknown	120	
Fluorescence quantum yields	0.14	Unknown	0.027	
Fluorescence engraftment rate	1.4	1	No engraftment	
Molecular target<br>Kd	VEGF<br>1.8-20 nM	$\alpha_v\beta_3$ integrin<br>3.9 nM	-	

The values were obtained from the Manufacturers.
